# Use of Chloroform and Ether

**Published:** 1862-04

**Authors:** 


### EDITORIAL DEPARTMENT.

### USE OF CHLOROFORM AND ETHER.

Discussions upon the comparative safety of chloroform and ether are reaching us from various quarters which are highly partisan, and in some instances almost personal in character. These discussions are highly interesting to every member of the profession, and all will feel a lively interest in arriving at last at proper and truthful conclusions. Opinions of men from their individual experience, or conclusions of societies in their corporate capacities are valuable and useful, and will contribute to the final settlement of this matter in the minds of many physicians; yet partisan bias and personal ambition if allowed place, will so warp the judgment and obscure the observation, that all value is lost, all correct opinion is destroyed, and we are left, after these discussions mainly as before, not persuaded that ether is perfectly safe and satisfactory in Boston, or chloroform in New York, more certain in its effects, much more agreeable, and fully as safe, perhaps safer than ether. We shall learn the number of deaths said to have been produced by chloroform and ether, and the disease which *might* have been present in each case, and to which death might have been attributed; but we shall hardly learn which of these two agents are likely to withstand the more rigid tests which are constantly being instituted concerning them. We shall not be likely to find in the end, that either one of these agents have been unanimously condemned as too unsafe to be used under any circumstances, or "perfectly safe and satisfactory."

The objection we wish to urge to the statements often used in this discussion are briefly these: too much is claimed and too little conceded, on both sides. Chloroform is probably in good hands, not so dangerous as its opponents would make us believe, and the responsibility of its administration is not so great as to deter surgeons from its use. Neither ether or chloroform are perfectly safe, but are potent, and dangerous, yet valuable agents, and in judicious, experienced hands, are both attended by so little risk and by so great certainty of relief from pain as to be proper agents for use whenever occasion demands; *provided always, that the dangers are understood, and that all due care be observed in the administration.*

For the last fourteen years we have used both ether and chloroform in a very large number of instances, both in private and hospital practice, in all ages, for all cases in which an anæsthetic is desirable, and to patients affected with various organic diseases, and thus far it has been our good fortune never to have seen any effects which would in the least, cause hesitation in administering, to a patient requiring it, either of these agents.

We do not desire to discuss their relative merits, at present, but to give our readers an opportunity of judging somewhat of the spirit often manifested, and thus to guard them from accepting any hasty conclusions which may be drawn upon either side of this long agitated question. The following editorial appears in the *Boston Medical and Surgical Journal,* which we copy for the benefit of our readers. While it commends itself in many respects to the consideration of physicians, it shows, we judge, that it is somewhat on the side of Boston. Boston is for ether; New York is partially at least on the side of chloroform. We most ardently desire for Buffalo, that it be only on the side of truth.

"DEATH FROM CHLOROFORM AT THE BELLEVUE HOSPITAL, NEW YORK. The occurrence of a death from chloroform in a metropolitan hospital, although the verdict of the coroner's jury exculpates the house-surgeon who administered it, suggests the inquiry whether the responsibility really devolving upon those who use so dangerous an agent can be thus readily shifted, and whether, in the light of present experience, it is not censurable, to use no stronger term, to expose patients, by the employment of one anæsthetic, to the risk of losing their lives, when in another we possess the means of securing an insensibility which is entirely satisfactory, and at the same time perfectly safe.

In the present instance chloroform was given to a young woman, admitted to the hospital on the 3d ult., for the purpose of manipulating a recent injury of the shoulder-joint; but a very small quantity was used, and that with every precaution, and the patient was not brought profoundly under its influence. She died before the examination was completed. We hear nothing against the quality of the article used; on the contrary, it is said to have been perfectly satisfactory. There was nothing unusual in the condition of the patient, the accident, or the circumstances of inhalation; it was a common case of death from chloroform, affording still another illustration of what all surgeons know, though some of them are reluctant to acknowledge the fact (witness the silence of the New York medical journals with regard to this case,) that no care taken, and no purity of the chloroform used, can avert the fatal shafts of this treacherous anæsthetic. It is very true, and fortunately so, that an event of this nature is of comparatively rare occurrence; that it happens for the first time in any particular hospital cannot, however, be claimed as a credit by its surgeons; they have only their good fortune to thank that so long a period of immunity in its use has been permitted them.

We learn that by a vote of the Surgeons of the New York Hospital, chloroform has not been used in that institution for twelve or fifteen years. A resolution formerly offered at the Bellevue Hospital that it should be administered only under the supervision of the attending physicians and surgeons, was not adopted. Such a recommendation now forms a part of the finding of the inquest. If the testimony of Dr. Willard Parker and Dr. James R. Wood in favor of ether is not to be heeded, it is to be hoped the suggestion of the jury will at least be listened to, and that the use of chloroform will henceforth be restricted to as few persons as possible. It is fortunate, perhaps, that the young gentlemen of the medical class of the Bellevue Hospital College, before being scattered to their homes, have had their attention forcibly called to the dangers of an agent which many of them, undoubtedly, have felt to be so innocuous in its nature. We are glad, too, if such occurrences, we can hardly call them accidents, must happen, that this one has preceded the vote about to be taken by the New York Academy of Medicine upon the conclusions of Dr. Barker's paper on the use of anæsthetics in midwifery. It cannot but make every member feel deeply the responsibility attaching itself to a vote which endorses the superiority of chloroform for any purpose whatever. Only the other day a correspondent of an English medical journal, writing from Paris, spoke of the hesitation with which chloroform was there given, and of the cries of the half-chloroformed patients undergoing operations by hospital surgeons who do not dare to withhold an anæsthetic, and yet give it with fear and trembling. It seems to us, if facts accumulate as they have heretofore, and men's minds remain open to conviction, and unbiased by prejudice or partisan feeling, that the responsibility attaching itself to the exhibition of chloroform, will soon be found here, as it bids fair to be in Paris, and (we are told by Dr. Hay, of this city, who has just returned from there) in Vienna also, greater than most surgeons will feel inclined to add to that of an operation.

Our readers will have anticipated us when we say that none of these apprehensions accompany the use of sulphuric ether, It is very easy to heap abuse upon the odor, or the bulk of this anæsthetic agent, or upon the zeal with which Boston defends its claims, and to assert that deaths have been caused by its inhalation. It is not so easy to point them out and prove them such. The burthen of proof rests with the friends and defenders of chloroform. That they have done this we are not aware. It is true that certain cases contained in the ether report of the Medical Improvement Society of this city have been quoted in a very general way against the committee, and one of their conclusions questioned; but no one, we believe, has undertaken to maintain, or show, that in any particular instance a fatal result was really and unquestionably due to the inhalation of pure sulphuric ether. The extent of objection has been that it is not made perfectly clear in certain cases that death *may* not have resulted from this cause. But without regard to the views of that committee, or the Society it represents, of the safety and entire efficiency of sulphuric ether there can be no doubt. Considerations of humanity, therefore, ask for a fair and candid trial of its claims to produce perfect anæsthesia, with a degree of security far superior to any other anæsthetic yet discovered; and we feel perfectly assured that any one who once becomes familiar with its use, will learn to place implicit confidence in the reliability of its properties."

